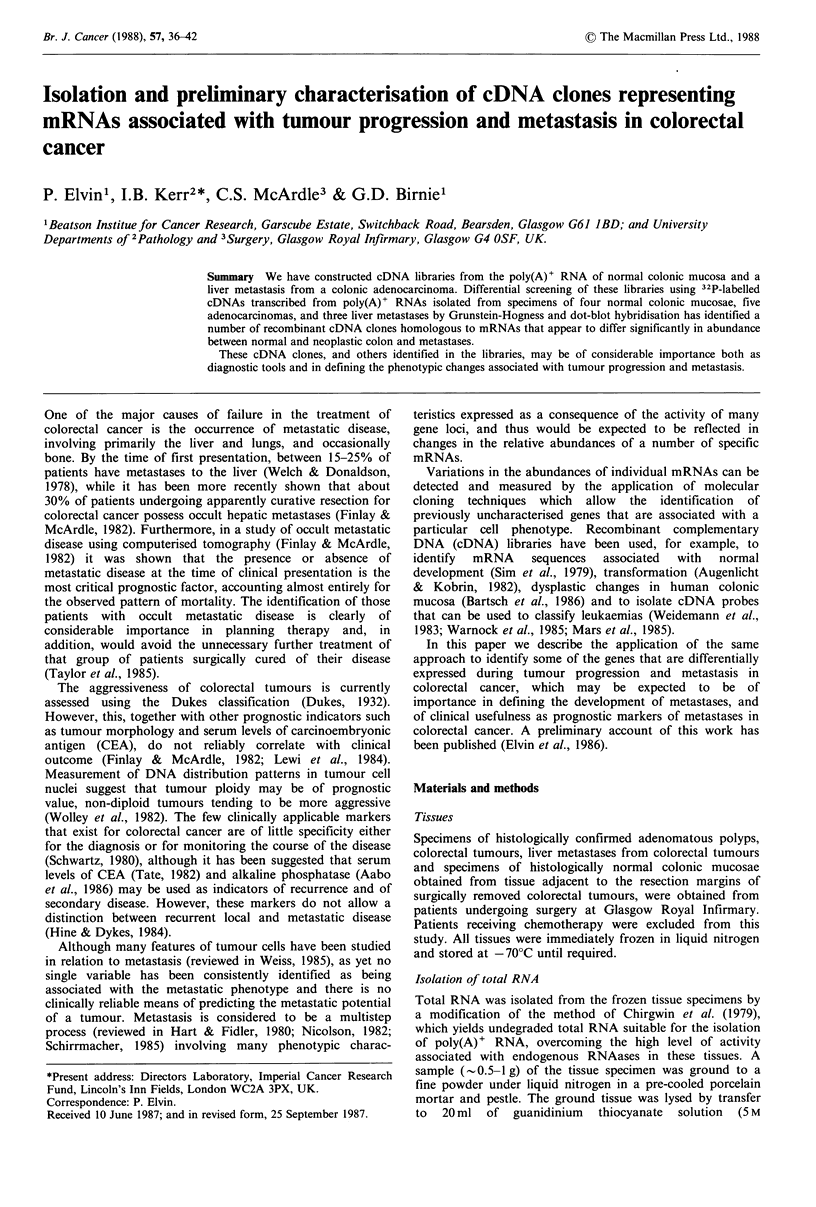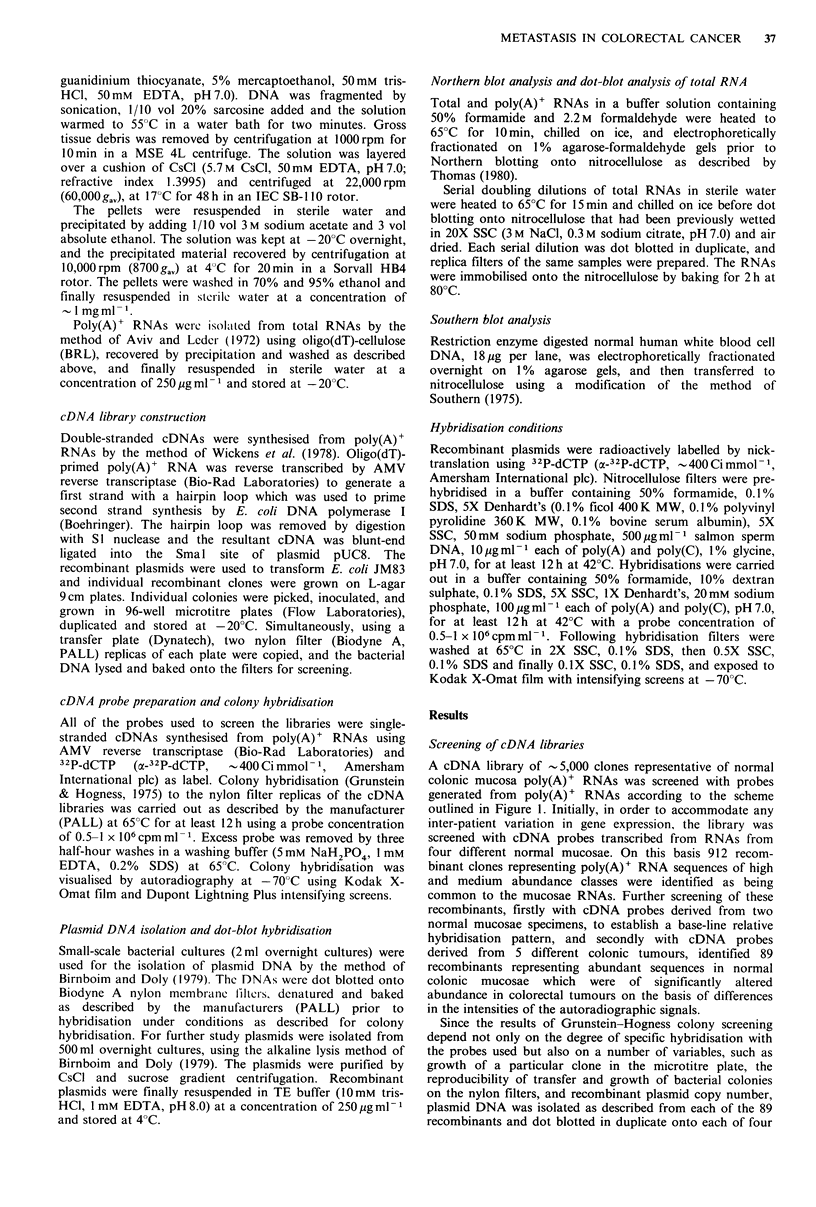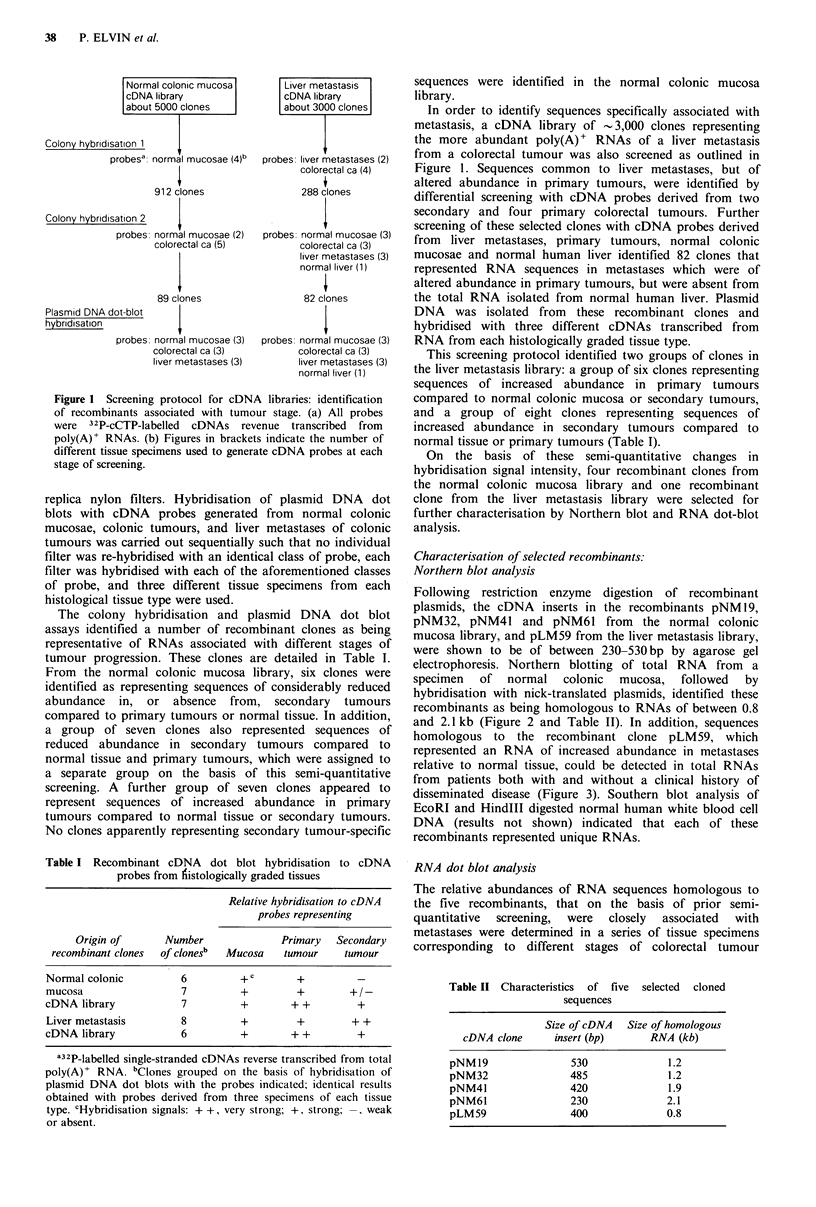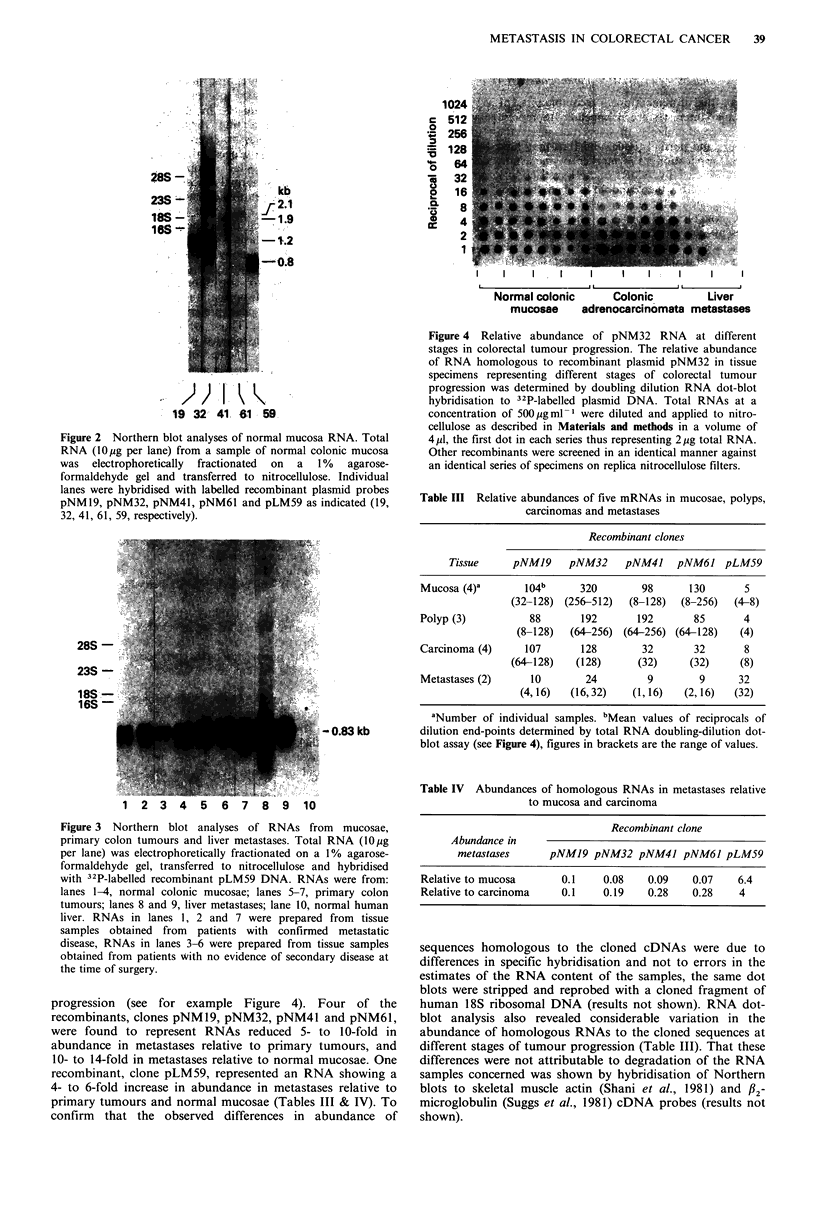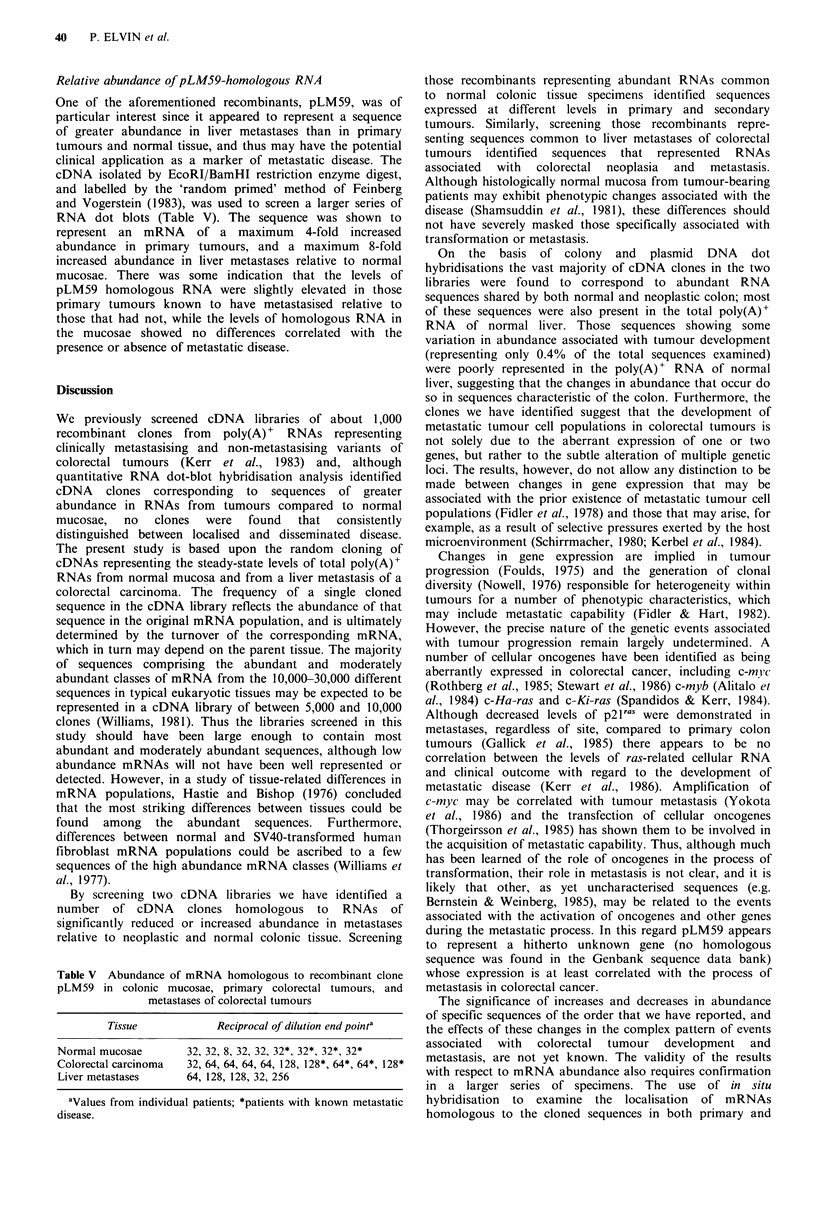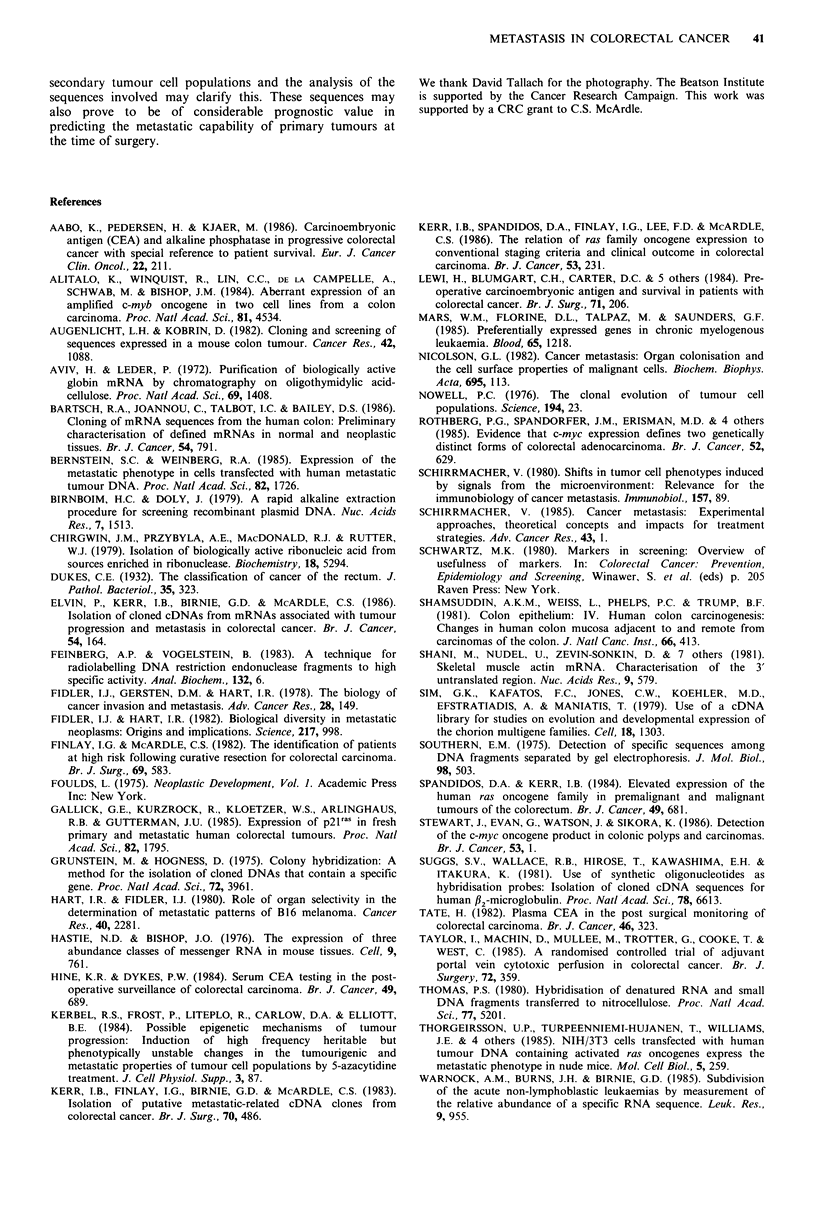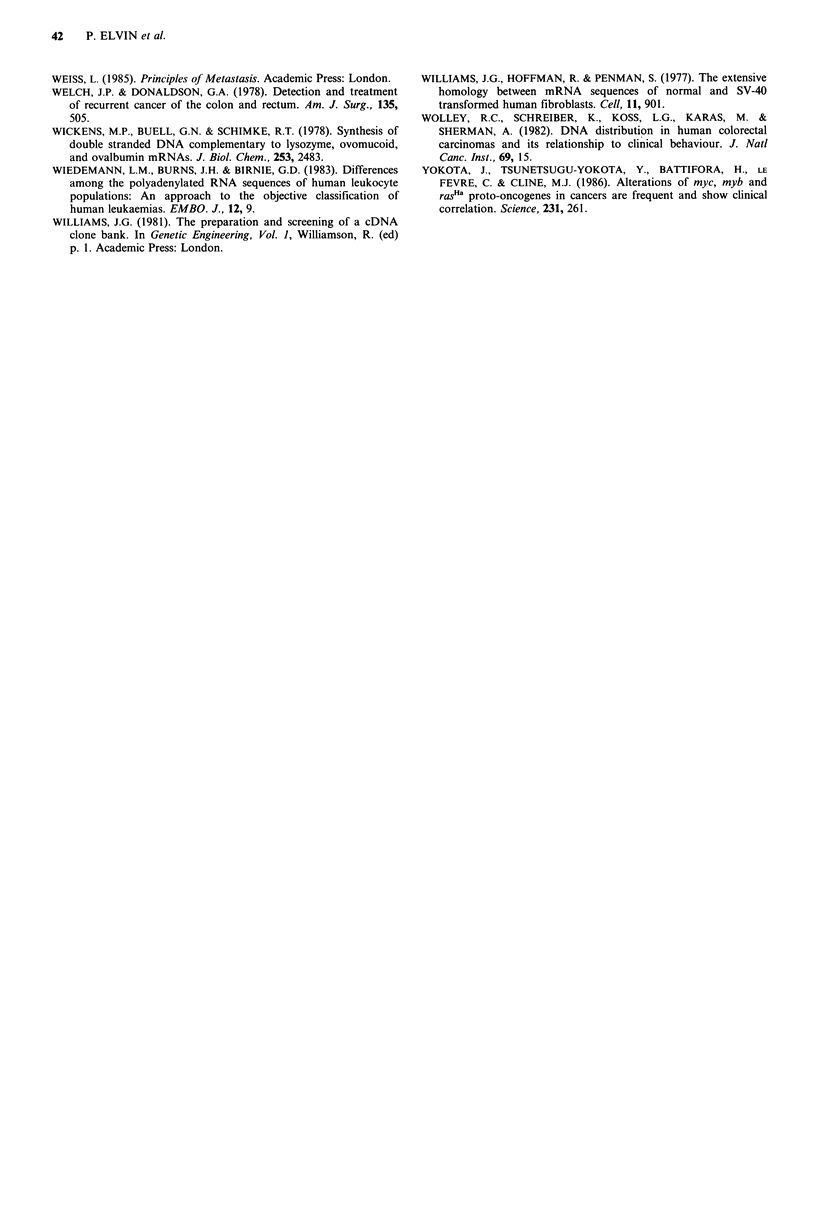# Isolation and preliminary characterisation of cDNA clones representing mRNAs associated with tumour progression and metastasis in colorectal cancer.

**DOI:** 10.1038/bjc.1988.5

**Published:** 1988-01

**Authors:** P. Elvin, I. B. Kerr, C. S. McArdle, G. D. Birnie

**Affiliations:** Beatson Institute for Cancer Research, Bearsden, Glasgow, UK.

## Abstract

**Images:**


*Br. J. Cancer* (1988), **57**, 36–42 © The Macmillan Press Ltd., 1988

### Isolation and preliminary characterisation of cDNA clones representing mRNAs associated with tumour progression and metastasis in colorectal cancer

P. Elvin[1], I.B. Kerr[2]*, C.S. McArdle[3] & G.D. Birnie[1]

[1]*Beatson Institue for Cancer Research, Garscube Estate, Switchback Road, Bearsden, Glasgow G61 1BD; and University Departments of* [2]*Pathology and* [3]*Surgery, Glasgow Royal Infirmary, Glasgow G4 0SF, UK.*

**Summary** We have constructed cDNA libraries from the poly(A)$^+$ RNA of normal colonic mucosa and a liver metastasis from a colonic adenocarcinoma. Differential screening of these libraries using $^{32}$P-labelled cDNAs transcribed from poly(A)$^+$ RNAs isolated from specimens of four normal colonic mucosae, five adenocarcinomas, and three liver metastases by Grunstein-Hogness and dot-blot hybridisation has identified a number of recombinant cDNA clones homologous to mRNAs that appear to differ significantly in abundance between normal and neoplastic colon and metastases.

These cDNA clones, and others identified in the libraries, may be of considerable importance both as diagnostic tools and in defining the phenotypic changes associated with tumour progression and metastasis.

One of the major causes of failure in the treatment of colorectal cancer is the occurrence of metastatic disease, involving primarily the liver and lungs, and occasionally bone. By the time of first presentation, between 15–25% of patients have metastases to the liver (Welch & Donaldson, 1978), while it has been more recently shown that about 30% of patients undergoing apparently curative resection for colorectal cancer possess occult hepatic metastases (Finlay & McArdle, 1982). Furthermore, in a study of occult metastatic disease using computerised tomography (Finlay & McArdle, 1982) it was shown that the presence or absence of metastatic disease at the time of clinical presentation is the most critical prognostic factor, accounting almost entirely for the observed pattern of mortality. The identification of those patients with occult metastatic disease is clearly of considerable importance in planning therapy and, in addition, would avoid the unnecessary further treatment of that group of patients surgically cured of their disease (Taylor *et al.*, 1985).

The aggressiveness of colorectal tumours is currently assessed using the Dukes classification (Dukes, 1932). However, this, together with other prognostic indicators such as tumour morphology and serum levels of carcinoembryonic antigen (CEA), do not reliably correlate with clinical outcome (Finlay & McArdle, 1982; Lewi *et al.*, 1984). Measurement of DNA distribution patterns in tumour cell nuclei suggest that tumour ploidy may be of prognostic value, non-diploid tumours tending to be more aggressive (Wolley *et al.*, 1982). The few clinically applicable markers that exist for colorectal cancer are of little specificity either for the diagnosis or for monitoring the course of the disease (Schwartz, 1980), although it has been suggested that serum levels of CEA (Tate, 1982) and alkaline phosphatase (Aabo *et al.*, 1986) may be used as indicators of recurrence and of secondary disease. However, these markers do not allow a distinction between recurrent local and metastatic disease (Hine & Dykes, 1984).

Although many features of tumour cells have been studied in relation to metastasis (reviewed in Weiss, 1985), as yet no single variable has been consistently identified as being associated with the metastatic phenotype and there is no clinically reliable means of predicting the metastatic potential of a tumour. Metastasis is considered to be a multistep process (reviewed in Hart & Fidler, 1980; Nicolson, 1982; Schirrmacher, 1985) involving many phenotypic characteristics expressed as a consequence of the activity of many gene loci, and thus would be expected to be reflected in changes in the relative abundances of a number of specific mRNAs.

Variations in the abundances of individual mRNAs can be detected and measured by the application of molecular cloning techniques which allow the identification of previously uncharacterised genes that are associated with a particular cell phenotype. Recombinant complementary DNA (cDNA) libraries have been used, for example, to identify mRNA sequences associated with normal development (Sim *et al.*, 1979), transformation (Augenlicht & Kobrin, 1982), dysplastic changes in human colonic mucosa (Bartsch *et al.*, 1986) and to isolate cDNA probes that can be used to classify leukaemias (Weidemann *et al.*, 1983; Warnock *et al.*, 1985; Mars *et al.*, 1985).

In this paper we describe the application of the same approach to identify some of the genes that are differentially expressed during tumour progression and metastasis in colorectal cancer, which may be expected to be of importance in defining the development of metastases, and of clinical usefulness as prognostic markers of metastases in colorectal cancer. A preliminary account of this work has been published (Elvin *et al.*, 1986).

### Materials and methods

### *Tissues*

Specimens of histologically confirmed adenomatous polyps, colorectal tumours, liver metastases from colorectal tumours and specimens of histologically normal colonic mucosae obtained from tissue adjacent to the resection margins of surgically removed colorectal tumours, were obtained from patients undergoing surgery at Glasgow Royal Infirmary. Patients receiving chemotherapy were excluded from this study. All tissues were immediately frozen in liquid nitrogen and stored at −70°C until required.

### *Isolation of total RNA*

Total RNA was isolated from the frozen tissue specimens by a modification of the method of Chirgwin *et al.* (1979), which yields undegraded total RNA suitable for the isolation of poly(A)$^+$ RNA, overcoming the high level of activity associated with endogenous RNAases in these tissues. A sample (~0.5–1 g) of the tissue specimen was ground to a fine powder under liquid nitrogen in a pre-cooled porcelain mortar and pestle. The ground tissue was lysed by transfer to 20 ml of guanidinium thiocyanate solution (5 M

*Present address: Directors Laboratory, Imperial Cancer Research Fund, Lincoln's Inn Fields, London WC2A 3PX, UK.
Correspondence: P. Elvin.
Received 10 June 1987; and in revised form, 25 September 1987.

guanidinium thiocyanate, 5% mercaptoethanol, 50 mM tris-HCl, 50 mM EDTA, pH 7.0). DNA was fragmented by sonication, 1/10 vol 20% sarcosine added and the solution warmed to 55°C in a water bath for two minutes. Gross tissue debris was removed by centrifugation at 1000 rpm for 10 min in a MSE 4L centrifuge. The solution was layered over a cushion of CsCl (5.7 M CsCl, 50 mM EDTA, pH 7.0; refractive index 1.3995) and centrifuged at 22,000 rpm (60,000 $g_{av}$), at 17°C for 48 h in an IEC SB-110 rotor.

The pellets were resuspended in sterile water and precipitated by adding 1/10 vol 3 M sodium acetate and 3 vol absolute ethanol. The solution was kept at −20°C overnight, and the precipitated material recovered by centrifugation at 10,000 rpm (8700 $g_{av}$) at 4°C for 20 min in a Sorvall HB4 rotor. The pellets were washed in 70% and 95% ethanol and finally resuspended in sterile water at a concentration of ~1 mg ml$^{-1}$.

Poly(A)$^+$ RNAs were isolated from total RNAs by the method of Aviv and Leder (1972) using oligo(dT)-cellulose (BRL), recovered by precipitation and washed as described above, and finally resuspended in sterile water at a concentration of 250 μg ml$^{-1}$ and stored at −20°C.

### *cDNA library construction*

Double-stranded cDNAs were synthesised from poly(A)$^+$ RNAs by the method of Wickens *et al.* (1978). Oligo(dT)-primed poly(A)$^+$ RNA was reverse transcribed by AMV reverse transcriptase (Bio-Rad Laboratories) to generate a first strand with a hairpin loop which was used to prime second strand synthesis by *E. coli* DNA polymerase I (Boehringer). The hairpin loop was removed by digestion with S1 nuclease and the resultant cDNA was blunt-end ligated into the SmaI site of plasmid pUC8. The recombinant plasmids were used to transform *E. coli* JM83 and individual recombinant clones were grown on L-agar 9 cm plates. Individual colonies were picked, inoculated, and grown in 96-well microtitre plates (Flow Laboratories), duplicated and stored at −20°C. Simultaneously, using a transfer plate (Dynatech), two nylon filter (Biodyne A, PALL) replicas of each plate were copied, and the bacterial DNA lysed and baked onto the filters for screening.

### *cDNA probe preparation and colony hybridisation*

All of the probes used to screen the libraries were single-stranded cDNAs synthesised from poly(A)$^+$ RNAs using AMV reverse transcriptase (Bio-Rad Laboratories) and $^{32}$P-dCTP ($\alpha$-$^{32}$P-dCTP, ~400 Ci mmol$^{-1}$, Amersham International plc) as label. Colony hybridisation (Grunstein & Hogness, 1975) to the nylon filter replicas of the cDNA libraries was carried out as described by the manufacturer (PALL) at 65°C for at least 12 h using a probe concentration of 0.5–1 x 10$^6$ cpm ml$^{-1}$. Excess probe was removed by three half-hour washes in a washing buffer (5 mM $NaH_2PO_4$, 1 mM EDTA, 0.2% SDS) at 65°C. Colony hybridisation was visualised by autoradiography at −70°C using Kodak X-Omat film and Dupont Lightning Plus intensifying screens.

### *Plasmid DNA isolation and dot-blot hybridisation*

Small-scale bacterial cultures (2 ml overnight cultures) were used for the isolation of plasmid DNA by the method of Birnboim and Doly (1979). The DNAs were dot blotted onto Biodyne A nylon membrane filters, denatured and baked as described by the manufacturers (PALL) prior to hybridisation under conditions as described for colony hybridisation. For further study plasmids were isolated from 500 ml overnight cultures, using the alkaline lysis method of Birnboim and Doly (1979). The plasmids were purified by CsCl and sucrose gradient centrifugation. Recombinant plasmids were finally resuspended in TE buffer (10 mM tris-HCl, 1 mM EDTA, pH 8.0) at a concentration of 250 μg ml$^{-1}$ and stored at 4°C.

### *Northern blot analysis and dot-blot analysis of total RNA*

Total and poly(A)$^+$ RNAs in a buffer solution containing 50% formamide and 2.2 M formaldehyde were heated to 65°C for 10 min, chilled on ice, and electrophoretically fractionated on 1% agarose-formaldehyde gels prior to Northern blotting onto nitrocellulose as described by Thomas (1980).

Serial doubling dilutions of total RNAs in sterile water were heated to 65°C for 15 min and chilled on ice before dot blotting onto nitrocellulose that had been previously wetted in 20X SSC (3 M NaCl, 0.3 M sodium citrate, pH 7.0) and air dried. Each serial dilution was dot blotted in duplicate, and replica filters of the same samples were prepared. The RNAs were immobilised onto the nitrocellulose by baking for 2 h at 80°C.

### *Southern blot analysis*

Restriction enzyme digested normal human white blood cell DNA, 18 μg per lane, was electrophoretically fractionated overnight on 1% agarose gels, and then transferred to nitrocellulose using a modification of the method of Southern (1975).

### *Hybridisation conditions*

Recombinant plasmids were radioactively labelled by nick-translation using $^{32}$P-dCTP ($\alpha$-$^{32}$P-dCTP, ~400 Ci mmol$^{-1}$, Amersham International plc). Nitrocellulose filters were pre-hybridised in a buffer containing 50% formamide, 0.1% SDS, 5X Denhardt's (0.1% ficol 400 K MW, 0.1% polyvinyl pyrolidine 360 K MW, 0.1% bovine serum albumin), 5X SSC, 50 mM sodium phosphate, 500 μg ml$^{-1}$ salmon sperm DNA, 10 μg ml$^{-1}$ each of poly(A) and poly(C), 1% glycine, pH 7.0, for at least 12 h at 42°C. Hybridisations were carried out in a buffer containing 50% formamide, 10% dextran sulphate, 0.1% SDS, 5X SSC, 1X Denhardt's, 20 mM sodium phosphate, 100 μg ml$^{-1}$ each of poly(A) and poly(C), pH 7.0, for at least 12 h at 42°C with a probe concentration of 0.5–1 x 10$^6$ cpm ml$^{-1}$. Following hybridisation filters were washed at 65°C in 2X SSC, 0.1% SDS, then 0.5X SSC, 0.1% SDS and finally 0.1X SSC, 0.1% SDS, and exposed to Kodak X-Omat film with intensifying screens at −70°C.

### Results

### *Screening of cDNA libraries*

A cDNA library of ~5,000 clones representative of normal colonic mucosa poly(A)$^+$ RNAs was screened with probes generated from poly(A)$^+$ RNAs according to the scheme outlined in Figure 1. Initially, in order to accommodate any inter-patient variation in gene expression, the library was screened with cDNA probes transcribed from RNAs from four different normal mucosae. On this basis 912 recombinant clones representing poly(A)$^+$ RNA sequences of high and medium abundance classes were identified as being common to the mucosae RNAs. Further screening of these recombinants, firstly with cDNA probes derived from two normal mucosae specimens, to establish a base-line relative hybridisation pattern, and secondly with cDNA probes derived from 5 different colonic tumours, identified 89 recombinants representing abundant sequences in normal colonic mucosae which were of significantly altered abundance in colorectal tumours on the basis of differences in the intensities of the autoradiographic signals.

Since the results of Grunstein–Hogness colony screening depend not only on the degree of specific hybridisation with the probes used but also on a number of variables, such as growth of a particular clone in the microtitre plate, the reproducibility of transfer and growth of bacterial colonies on the nylon filters, and recombinant plasmid copy number, plasmid DNA was isolated as described from each of the 89 recombinants and dot blotted in duplicate onto each of four

**Figure 1** Screening protocol for cDNA libraries: identification of recombinants associated with tumour stage. (a) All probes were $^{32}$P-cCTP-labelled cDNAs revenue transcribed from poly(A)$^+$ RNAs. (b) Figures in brackets indicate the number of different tissue specimens used to generate cDNA probes at each stage of screening.

replica nylon filters. Hybridisation of plasmid DNA dot blots with cDNA probes generated from normal colonic mucosae, colonic tumours, and liver metastases of colonic tumours was carried out sequentially such that no individual filter was re-hybridised with an identical class of probe, each filter was hybridised with each of the aforementioned classes of probe, and three different tissue specimens from each histological tissue type were used.

The colony hybridisation and plasmid DNA dot blot assays identified a number of recombinant clones as being representative of RNAs associated with different stages of tumour progression. These clones are detailed in Table I. From the normal colonic mucosa library, six clones were identified as representing sequences of considerably reduced abundance in, or absence from, secondary tumours compared to primary tumours or normal tissue. In addition, a group of seven clones also represented sequences of reduced abundance in secondary tumours compared to normal tissue and primary tumours, which were assigned to a separate group on the basis of this semi-quantitative screening. A further group of seven clones appeared to represent sequences of increased abundance in primary tumours compared to normal tissue or secondary tumours. No clones apparently representing secondary tumour-specific

**Table I** Recombinant cDNA dot blot hybridisation to cDNA probes from histologically graded tissues

| | | *Relative hybridisation to cDNA probes representing* | | |
|---|---|---|---|---|
| *Origin of recombinant clones* | *Number of clones*[b] | *Mucosa* | *Primary tumour* | *Secondary tumour* |
| Normal colonic | 6 | +[c] | + | − |
| mucosa | 7 | + | + | +/− |
| cDNA library | 7 | + | ++ | + |
| Liver metastasis | 8 | + | + | ++ |
| cDNA library | 6 | + | ++ | + |

[a] $^{32}$P-labelled single-stranded cDNAs reverse transcribed from total poly(A)$^+$ RNA. [b] Clones grouped on the basis of hybridisation of plasmid DNA dot blots with the probes indicated; identical results obtained with probes derived from three specimens of each tissue type. [c] Hybridisation signals: ++, very strong; +, strong; −, weak or absent.

sequences were identified in the normal colonic mucosa library.

In order to identify sequences specifically associated with metastasis, a cDNA library of ~3,000 clones representing the more abundant poly(A)$^+$ RNAs of a liver metastasis from a colorectal tumour was also screened as outlined in Figure 1. Sequences common to liver metastases, but of altered abundance in primary tumours, were identified by differential screening with cDNA probes derived from two secondary and four primary colorectal tumours. Further screening of these selected clones with cDNA probes derived from liver metastases, primary tumours, normal colonic mucosae and normal human liver identified 82 clones that represented RNA sequences in metastases which were of altered abundance in primary tumours, but were absent from the total RNA isolated from normal human liver. Plasmid DNA was isolated from these recombinant clones and hybridised with three different cDNAs transcribed from RNA from each histologically graded tissue type.

This screening protocol identified two groups of clones in the liver metastasis library: a group of six clones representing sequences of increased abundance in primary tumours compared to normal colonic mucosa or secondary tumours, and a group of eight clones representing sequences of increased abundance in secondary tumours compared to normal tissue or primary tumours (Table I).

On the basis of these semi-quantitative changes in hybridisation signal intensity, four recombinant clones from the normal colonic mucosa library and one recombinant clone from the liver metastasis library were selected for further characterisation by Northern blot and RNA dot-blot analysis.

### *Characterisation of selected recombinants: Northern blot analysis*

Following restriction enzyme digestion of recombinant plasmids, the cDNA inserts in the recombinants pNM19, pNM32, pNM41 and pNM61 from the normal colonic mucosa library, and pLM59 from the liver metastasis library, were shown to be of between 230–530 bp by agarose gel electrophoresis. Northern blotting of total RNA from a specimen of normal colonic mucosa, followed by hybridisation with nick-translated plasmids, identified these recombinants as being homologous to RNAs of between 0.8 and 2.1 kb (Figure 2 and Table II). In addition, sequences homologous to the recombinant clone pLM59, which represented an RNA of increased abundance in metastases relative to normal tissue, could be detected in total RNAs from patients both with and without a clinical history of disseminated disease (Figure 3). Southern blot analysis of EcoRI and HindIII digested normal human white blood cell DNA (results not shown) indicated that each of these recombinants represented unique RNAs.

### *RNA dot blot analysis*

The relative abundances of RNA sequences homologous to the five recombinants, that on the basis of prior semi-quantitative screening, were closely associated with metastases were determined in a series of tissue specimens corresponding to different stages of colorectal tumour

**Table II** Characteristics of five selected cloned sequences

| *cDNA clone* | *Size of cDNA insert (bp)* | *Size of homologous RNA (kb)* |
|---|---|---|
| pNM19 | 530 | 1.2 |
| pNM32 | 485 | 1.2 |
| pNM41 | 420 | 1.9 |
| pNM61 | 230 | 2.1 |
| pLM59 | 400 | 0.8 |

**Figure 2** Northern blot analyses of normal mucosa RNA. Total RNA (10 μg per lane) from a sample of normal colonic mucosa was electrophoretically fractionated on a 1% agarose-formaldehyde gel and transferred to nitrocellulose. Individual lanes were hybridised with labelled recombinant plasmid probes pNM19, pNM32, pNM41, pNM61 and pLM59 as indicated (19, 32, 41, 61, 59, respectively).

**Figure 3** Northern blot analyses of RNAs from mucosae, primary colon tumours and liver metastases. Total RNA (10 μg per lane) was electrophoretically fractionated on a 1% agarose-formaldehyde gel, transferred to nitrocellulose and hybridised with $^{32}P$-labelled recombinant pLM59 DNA. RNAs were from: lanes 1–4, normal colonic mucosae; lanes 5–7, primary colon tumours; lanes 8 and 9, liver metastases; lane 10, normal human liver. RNAs in lanes 1, 2 and 7 were prepared from tissue samples obtained from patients with confirmed metastatic disease, RNAs in lanes 3–6 were prepared from tissue samples obtained from patients with no evidence of secondary disease at the time of surgery.

progression (see for example Figure 4). Four of the recombinants, clones pNM19, pNM32, pNM41 and pNM61, were found to represent RNAs reduced 5- to 10-fold in abundance in metastases relative to primary tumours, and 10- to 14-fold in metastases relative to normal mucosae. One recombinant, clone pLM59, represented an RNA showing a 4- to 6-fold increase in abundance in metastases relative to primary tumours and normal mucosae (Tables III & IV). To confirm that the observed differences in abundance of

**Figure 4** Relative abundance of pNM32 RNA at different stages in colorectal tumour progression. The relative abundance of RNA homologous to recombinant plasmid pNM32 in tissue specimens representing different stages of colorectal tumour progression was determined by doubling dilution RNA dot-blot hybridisation to $^{32}P$-labelled plasmid DNA. Total RNAs at a concentration of $500\,\mu g\,ml^{-1}$ were diluted and applied to nitrocellulose as described in **Materials and methods** in a volume of 4 μl, the first dot in each series thus representing 2 μg total RNA. Other recombinants were screened in an identical manner against an identical series of specimens on replica nitrocellulose filters.

**Table III** Relative abundances of five mRNAs in mucosae, polyps, carcinomas and metastases

| *Tissue* | *Recombinant clones* pNM19 | *pNM32* | *pNM41* | *pNM61* | *pLM59* |
|---|---|---|---|---|---|
| Mucosa (4)[a] | 104[b] (32–128) | 320 (256–512) | 98 (8–128) | 130 (8–256) | 5 (4–8) |
| Polyp (3) | 88 (8–128) | 192 (64–256) | 192 (64–256) | 85 (64–128) | 4 (4) |
| Carcinoma (4) | 107 (64–128) | 128 (128) | 32 (32) | 32 (32) | 8 (8) |
| Metastases (2) | 10 (4, 16) | 24 (16, 32) | 9 (1, 16) | 9 (2, 16) | 32 (32) |

[a]Number of individual samples. [b]Mean values of reciprocals of dilution end-points determined by total RNA doubling-dilution dot-blot assay (see **Figure 4**), figures in brackets are the range of values.

**Table IV** Abundances of homologous RNAs in metastases relative to mucosa and carcinoma

| *Abundance in metastases* | *Recombinant clone* pNM19 | *pNM32* | *pNM41* | *pNM61* | *pLM59* |
|---|---|---|---|---|---|
| Relative to mucosa | 0.1 | 0.08 | 0.09 | 0.07 | 6.4 |
| Relative to carcinoma | 0.1 | 0.19 | 0.28 | 0.28 | 4 |

sequences homologous to the cloned cDNAs were due to differences in specific hybridisation and not to errors in the estimates of the RNA content of the samples, the same dot blots were stripped and reprobed with a cloned fragment of human 18S ribosomal DNA (results not shown). RNA dot-blot analysis also revealed considerable variation in the abundance of homologous RNAs to the cloned sequences at different stages of tumour progression (Table III). That these differences were not attributable to degradation of the RNA samples concerned was shown by hybridisation of Northern blots to skeletal muscle actin (Shani *et al.*, 1981) and $\beta_2$-microglobulin (Suggs *et al.*, 1981) cDNA probes (results not shown).

*Relative abundance of pLM59-homologous RNA*

One of the aforementioned recombinants, pLM59, was of particular interest since it appeared to represent a sequence of greater abundance in liver metastases than in primary tumours and normal tissue, and thus may have the potential clinical application as a marker of metastatic disease. The cDNA isolated by EcoRI/BamHI restriction enzyme digest, and labelled by the 'random primed' method of Feinberg and Vogerstein (1983), was used to screen a larger series of RNA dot blots (Table V). The sequence was shown to represent an mRNA of a maximum 4-fold increased abundance in primary tumours, and a maximum 8-fold increased abundance in liver metastases relative to normal mucosae. There was some indication that the levels of pLM59 homologous RNA were slightly elevated in those primary tumours known to have metastasised relative to those that had not, while the levels of homologous RNA in the mucosae showed no differences correlated with the presence or absence of metastatic disease.

### Discussion

We previously screened cDNA libraries of about 1,000 recombinant clones from poly(A)$^+$ RNAs representing clinically metastasising and non-metastasising variants of colorectal tumours (Kerr *et al.*, 1983) and, although quantitative RNA dot-blot hybridisation analysis identified cDNA clones corresponding to sequences of greater abundance in RNAs from tumours compared to normal mucosae, no clones were found that consistently distinguished between localised and disseminated disease. The present study is based upon the random cloning of cDNAs representing the steady-state levels of total poly(A)$^+$ RNAs from normal mucosa and from a liver metastasis of a colorectal carcinoma. The frequency of a single cloned sequence in the cDNA library reflects the abundance of that sequence in the original mRNA population, and is ultimately determined by the turnover of the corresponding mRNA, which in turn may depend on the parent tissue. The majority of sequences comprising the abundant and moderately abundant classes of mRNA from the 10,000–30,000 different sequences in typical eukaryotic tissues may be expected to be represented in a cDNA library of between 5,000 and 10,000 clones (Williams, 1981). Thus the libraries screened in this study should have been large enough to contain most abundant and moderately abundant sequences, although low abundance mRNAs will not have been well represented or detected. However, in a study of tissue-related differences in mRNA populations, Hastie and Bishop (1976) concluded that the most striking differences between tissues could be found among the abundant sequences. Furthermore, differences between normal and SV40-transformed human fibroblast mRNA populations could be ascribed to a few sequences of the high abundance mRNA classes (Williams *et al.*, 1977).

By screening two cDNA libraries we have identified a number of cDNA clones homologous to RNAs of significantly reduced or increased abundance in metastases relative to neoplastic and normal colonic tissue. Screening those recombinants representing abundant RNAs common to normal colonic tissue specimens identified sequences expressed at different levels in primary and secondary tumours. Similarly, screening those recombinants representing sequences common to liver metastases of colorectal tumours identified sequences that represented RNAs associated with colorectal neoplasia and metastasis. Although histologically normal mucosa from tumour-bearing patients may exhibit phenotypic changes associated with the disease (Shamsuddin *et al.*, 1981), these differences should not have severely masked those specifically associated with transformation or metastasis.

On the basis of colony and plasmid DNA dot hybridisations the vast majority of cDNA clones in the two libraries were found to correspond to abundant RNA sequences shared by both normal and neoplastic colon; most of these sequences were also present in the total poly(A)$^+$ RNA of normal liver. Those sequences showing some variation in abundance associated with tumour development (representing only 0.4% of the total sequences examined) were poorly represented in the poly(A)$^+$ RNA of normal liver, suggesting that the changes in abundance that occur do so in sequences characteristic of the colon. Furthermore, the clones we have identified suggest that the development of metastatic tumour cell populations in colorectal tumours is not solely due to the aberrant expression of one or two genes, but rather to the subtle alteration of multiple genetic loci. The results, however, do not allow any distinction to be made between changes in gene expression that may be associated with the prior existence of metastatic tumour cell populations (Fidler *et al.*, 1978) and those that may arise, for example, as a result of selective pressures exerted by the host microenvironment (Schirrmacher, 1980; Kerbel *et al.*, 1984).

Changes in gene expression are implied in tumour progression (Foulds, 1975) and the generation of clonal diversity (Nowell, 1976) responsible for heterogeneity within tumours for a number of phenotypic characteristics, which may include metastatic capability (Fidler & Hart, 1982). However, the precise nature of the genetic events associated with tumour progression remain largely undetermined. A number of cellular oncogenes have been identified as being aberrantly expressed in colorectal cancer, including c-*myc* (Rothberg *et al.*, 1985; Stewart *et al.*, 1986) c-*myb* (Alitalo *et al.*, 1984) c-*Ha-ras* and c-*Ki-ras* (Spandidos & Kerr, 1984). Although decreased levels of $p21^{ras}$ were demonstrated in metastases, regardless of site, compared to primary colon tumours (Gallick *et al.*, 1985) there appears to be no correlation between the levels of *ras*-related cellular RNA and clinical outcome with regard to the development of metastatic disease (Kerr *et al.*, 1986). Amplification of c-*myc* may be correlated with tumour metastasis (Yokota *et al.*, 1986) and the transfection of cellular oncogenes (Thorgeirsson *et al.*, 1985) has shown them to be involved in the acquisition of metastatic capability. Thus, although much has been learned of the role of oncogenes in the process of transformation, their role in metastasis is not clear, and it is likely that other, as yet uncharacterised sequences (e.g. Bernstein & Weinberg, 1985), may be related to the events associated with the activation of oncogenes and other genes during the metastatic process. In this regard pLM59 appears to represent a hitherto unknown gene (no homologous sequence was found in the Genbank sequence data bank) whose expression is at least correlated with the process of metastasis in colorectal cancer.

The significance of increases and decreases in abundance of specific sequences of the order that we have reported, and the effects of these changes in the complex pattern of events associated with colorectal tumour development and metastasis, are not yet known. The validity of the results with respect to mRNA abundance also requires confirmation in a larger series of specimens. The use of *in situ* hybridisation to examine the localisation of mRNAs homologous to the cloned sequences in both primary and

**Table V** Abundance of mRNA homologous to recombinant clone pLM59 in colonic mucosae, primary colorectal tumours, and metastases of colorectal tumours

| *Tissue* | *Reciprocal of dilution end point*[a] |
|---|---|
| Normal mucosae | 32, 32, 8, 32, 32, 32*, 32*, 32*, 32* |
| Colorectal carcinoma | 32, 64, 64, 64, 64, 128, 128*, 64*, 64*, 128* |
| Liver metastases | 64, 128, 128, 32, 256 |

[a]Values from individual patients; *patients with known metastatic disease.

secondary tumour cell populations and the analysis of the sequences involved may clarify this. These sequences may also prove to be of considerable prognostic value in predicting the metastatic capability of primary tumours at the time of surgery.

We thank David Tallach for the photography. The Beatson Institute is supported by the Cancer Research Campaign. This work was supported by a CRC grant to C.S. McArdle.

### References

AABO, K., PEDERSEN, H. & KJAER, M. (1986). Carcinoembryonic antigen (CEA) and alkaline phosphatase in progressive colorectal cancer with special reference to patient survival. *Eur. J. Cancer Clin. Oncol.*, **22**, 211.

ALITALO, K., WINQUIST, R., LIN, C.C., DE LA CAMPELLE, A., SCHWAB, M. & BISHOP, J.M. (1984). Aberrant expression of an amplified c-*myb* oncogene in two cell lines from a colon carcinoma. *Proc. Natl Acad. Sci.*, **81**, 4534.

AUGENLICHT, L.H. & KOBRIN, D. (1982). Cloning and screening of sequences expressed in a mouse colon tumour. *Cancer Res.*, **42**, 1088.

AVIV, H. & LEDER, P. (1972). Purification of biologically active globin mRNA by chromatography on oligothymidylic acid-cellulose. *Proc. Natl Acad. Sci.*, **69**, 1408.

BARTSCH, R.A., JOANNOU, C., TALBOT, I.C. & BAILEY, D.S. (1986). Cloning of mRNA sequences from the human colon: Preliminary characterisation of defined mRNAs in normal and neoplastic tissues. *Br. J. Cancer*, **54**, 791.

BERNSTEIN, S.C. & WEINBERG, R.A. (1985). Expression of the metastatic phenotype in cells transfected with human metastatic tumour DNA. *Proc. Natl Acad. Sci.*, **82**, 1726.

BIRNBOIM, H.C. & DOLY, J. (1979). A rapid alkaline extraction procedure for screening recombinant plasmid DNA. *Nuc. Acids Res.*, **7**, 1513.

CHIRGWIN, J.M., PRZYBYLA, A.E., MACDONALD, R.J. & RUTTER, W.J. (1979). Isolation of biologically active ribonucleic acid from sources enriched in ribonuclease. *Biochemistry*, **18**, 5294.

DUKES, C.E. (1932). The classification of cancer of the rectum. *J. Pathol. Bacteriol.*, **35**, 323.

ELVIN, P., KERR, I.B., BIRNIE, G.D. & MCARDLE, C.S. (1986). Isolation of cloned cDNAs from mRNAs associated with tumour progression and metastasis in colorectal cancer. *Br. J. Cancer*, **54**, 164.

FEINBERG, A.P. & VOGELSTEIN, B. (1983). A technique for radiolabelling DNA restriction endonuclease fragments to high specific activity. *Anal. Biochem.*, **132**, 6.

FIDLER, I.J., GERSTEN, D.M. & HART, I.R. (1978). The biology of cancer invasion and metastasis. *Adv. Cancer Res.*, **28**, 149.

FIDLER, I.J. & HART, I.R. (1982). Biological diversity in metastatic neoplasms: Origins and implications. *Science*, **217**, 998.

FINLAY, I.G. & MCARDLE, C.S. (1982). The identification of patients at high risk following curative resection for colorectal carcinoma. *Br. J. Surg.*, **69**, 583.

FOULDS, L. (1975). *Neoplastic Development, Vol. 1.* Academic Press Inc: New York.

GALLICK, G.E., KURZROCK, R., KLOETZER, W.S., ARLINGHAUS, R.B. & GUTTERMAN, J.U. (1985). Expression of $p21^{ras}$ in fresh primary and metastatic human colorectal tumours. *Proc. Natl Acad. Sci.*, **82**, 1795.

GRUNSTEIN, M. & HOGNESS, D. (1975). Colony hybridization: A method for the isolation of cloned DNAs that contain a specific gene. *Proc. Natl Acad. Sci.*, **72**, 3961.

HART, I.R. & FIDLER, I.J. (1980). Role of organ selectivity in the determination of metastatic patterns of B16 melanoma. *Cancer Res.*, **40**, 2281.

HASTIE, N.D. & BISHOP, J.O. (1976). The expression of three abundance classes of messenger RNA in mouse tissues. *Cell*, **9**, 761.

HINE, K.R. & DYKES, P.W. (1984). Serum CEA testing in the post-operative surveillance of colorectal carcinoma. *Br. J. Cancer*, **49**, 689.

KERBEL, R.S., FROST, P., LITEPLO, R., CARLOW, D.A. & ELLIOTT, B.E. (1984). Possible epigenetic mechanisms of tumour progression: Induction of high frequency heritable but phenotypically unstable changes in the tumourigenic and metastatic properties of tumour cell populations by 5-azacytidine treatment. *J. Cell Physiol. Supp.*, **3**, 87.

KERR, I.B., FINLAY, I.G., BIRNIE, G.D. & MCARDLE, C.S. (1983). Isolation of putative metastatic-related cDNA clones from colorectal cancer. *Br. J. Surg.*, **70**, 486.

KERR, I.B., SPANDIDOS, D.A., FINLAY, I.G., LEE, F.D. & MCARDLE, C.S. (1986). The relation of *ras* family oncogene expression to conventional staging criteria and clinical outcome in colorectal carcinoma. *Br. J. Cancer*, **53**, 231.

LEWI, H., BLUMGART, C.H., CARTER, D.C. & 5 others (1984). Pre-operative carcinoembryonic antigen and survival in patients with colorectal cancer. *Br. J. Surg.*, **71**, 206.

MARS, W.M., FLORINE, D.L., TALPAZ, M. & SAUNDERS, G.F. (1985). Preferentially expressed genes in chronic myelogenous leukaemia. *Blood*, **65**, 1218.

NICOLSON, G.L. (1982). Cancer metastasis: Organ colonisation and the cell surface properties of malignant cells. *Biochem. Biophys. Acta*, **695**, 113.

NOWELL, P.C. (1976). The clonal evolution of tumour cell populations. *Science*, **194**, 23.

ROTHBERG, P.G., SPANDORFER, J.M., ERISMAN, M.D. & 4 others (1985). Evidence that c-*myc* expression defines two genetically distinct forms of colorectal adenocarcinoma. *Br. J. Cancer*, **52**, 629.

SCHIRRMACHER, V. (1980). Shifts in tumor cell phenotypes induced by signals from the microenvironment: Relevance for the immunobiology of cancer metastasis. *Immunobiol.*, **157**, 89.

SCHIRRMACHER, V. (1985). Cancer metastasis: Experimental approaches, theoretical concepts and impacts for treatment strategies. *Adv. Cancer Res.*, **43**, 1.

SCHWARTZ, M.K. (1980). Markers in screening: Overview of usefulness of markers. In: *Colorectal Cancer: Prevention, Epidemiology and Screening*, Winawer, S. *et al.* (eds) p. 205 Raven Press: New York.

SHAMSUDDIN, A.K.M., WEISS, L., PHELPS, P.C. & TRUMP, B.F. (1981). Colon epithelium: IV. Human colon carcinogenesis: Changes in human colon mucosa adjacent to and remote from carcinomas of the colon. *J. Natl Canc. Inst.*, **66**, 413.

SHANI, M., NUDEL, U., ZEVIN-SONKIN, D. & 7 others (1981). Skeletal muscle actin mRNA. Characterisation of the 3′ untranslated region. *Nuc. Acids Res.*, **9**, 579.

SIM, G.K., KAFATOS, F.C., JONES, C.W., KOEHLER, M.D., EFSTRATIADIS, A. & MANIATIS, T. (1979). Use of a cDNA library for studies on evolution and developmental expression of the chorion multigene families. *Cell*, **18**, 1303.

SOUTHERN, E.M. (1975). Detection of specific sequences among DNA fragments separated by gel electrophoresis. *J. Mol. Biol.*, **98**, 503.

SPANDIDOS, D.A. & KERR, I.B. (1984). Elevated expression of the human *ras* oncogene family in premalignant and malignant tumours of the colorectum. *Br. J. Cancer*, **49**, 681.

STEWART, J., EVAN, G., WATSON, J. & SIKORA, K. (1986). Detection of the c-*myc* oncogene product in colonic polyps and carcinomas. *Br. J. Cancer*, **53**, 1.

SUGGS, S.V., WALLACE, R.B., HIROSE, T., KAWASHIMA, E.H. & ITAKURA, K. (1981). Use of synthetic oligonucleotides as hybridisation probes: Isolation of cloned cDNA sequences for human $\beta_2$-microglobulin. *Proc. Natl Acad. Sci.*, **78**, 6613.

TATE, H. (1982). Plasma CEA in the post surgical monitoring of colorectal carcinoma. *Br. J. Cancer*, **46**, 323.

TAYLOR, I., MACHIN, D., MULLEE, M., TROTTER, G., COOKE, T. & WEST, C. (1985). A randomised controlled trial of adjuvant portal vein cytotoxic perfusion in colorectal cancer. *Br. J. Surgery*, **72**, 359.

THOMAS, P.S. (1980). Hybridisation of denatured RNA and small DNA fragments transferred to nitrocellulose. *Proc. Natl Acad. Sci.*, **77**, 5201.

THORGEIRSSON, U.P., TURPEENNIEMI-HUJANEN, T., WILLIAMS, J.E. & 4 others (1985). NIH/3T3 cells transfected with human tumour DNA containing activated *ras* oncogenes express the metastatic phenotype in nude mice. *Mol. Cell Biol.*, **5**, 259.

WARNOCK, A.M., BURNS, J.H. & BIRNIE, G.D. (1985). Subdivision of the acute non-lymphoblastic leukaemias by measurement of the relative abundance of a specific RNA sequence. *Leuk. Res.*, **9**, 955.

WEISS, L. (1985). *Principles of Metastasis*. Academic Press: London.

WELCH, J.P. & DONALDSON, G.A. (1978). Detection and treatment of recurrent cancer of the colon and rectum. *Am. J. Surg.*, **135**, 505.

WICKENS, M.P., BUELL, G.N. & SCHIMKE, R.T. (1978). Synthesis of double stranded DNA complementary to lysozyme, ovomucoid, and ovalbumin mRNAs. *J. Biol. Chem.*, **253**, 2483.

WIEDEMANN, L.M., BURNS, J.H. & BIRNIE, G.D. (1983). Differences among the polyadenylated RNA sequences of human leukocyte populations: An approach to the objective classification of human leukaemias. *EMBO. J.*, **12**, 9.

WILLIAMS, J.G. (1981). The preparation and screening of a cDNA clone bank. In *Genetic Engineering, Vol. 1*, Williamson, R. (ed) p. 1. Academic Press: London.

WILLIAMS, J.G., HOFFMAN, R. & PENMAN, S. (1977). The extensive homology between mRNA sequences of normal and SV-40 transformed human fibroblasts. *Cell*, **11**, 901.

WOLLEY, R.C., SCHREIBER, K., KOSS, L.G., KARAS, M. & SHERMAN, A. (1982). DNA distribution in human colorectal carcinomas and its relationship to clinical behaviour. *J. Natl Canc. Inst.*, **69**, 15.

YOKOTA, J., TSUNETSUGU-YOKOTA, Y., BATTIFORA, H., LE FEVRE, C. & CLINE, M.J. (1986). Alterations of *myc*, *myb* and *ras*$^{Ha}$ proto-oncogenes in cancers are frequent and show clinical correlation. *Science*, **231**, 261.